# High Mechanical and Thermal Properties of Epoxy Composites with Liquid Crystalline Polyurethane Modified Graphene

**DOI:** 10.3390/polym10050485

**Published:** 2018-05-01

**Authors:** Yuqi Li, Jian Gao, Xiuyun Li, Xu Xu, Shaorong Lu

**Affiliations:** Key Laboratory of New Processing Technology for Nonferrous Metals and Materials, Ministry of Education, College of Materials Science and Engineering, Guilin University of Technology, Guilin 541004, China; gaojian1633@163.com (J.G.); 18577392856@163.com (X.L.); xuxu8485@163.com (X.X.)

**Keywords:** graphene, liquid crystalline polyurethane, epoxy resin, composites

## Abstract

Graphene nanosheets (GNs) often result in incompatibility with the hydrophobic polymer matrix, and the tendency to form aggregates during processing. Herein, liquid crystalline polyurethane modified GNs (GPLP) were obtained by π–π stacking interactions between GNs and perylene bisimide derivatives, and then in-situ polymerization of liquid-crystalline polyurethane. Spectroscopic studies, elemental analysis, and thermal properties confirmed the successful π–π stacking and the integrated structure of GPLP. The good dispersion state of GPLP in the epoxy matrix (EP), and the strong interactions between GPLP and EP, lead to the significant improvement of the thermal and mechanical performance of the GPLP/EP composites. The impact strength, Young’s modulus, tensile strength, and toughness of the GPLP/EP composites with 1.47 wt % GNs reached the highest values of 54.31 kJ/m^2^, 530.8 MPa, 112.33 MPa and 863 J/m^3^, which significantly increased by 210%, 57%, 143%, and 122% compared to that of neat epoxy, respectively. As well, the glass transition temperature increased by a notable 33 °C. It is hoped that this work can be used to exploit more efficient methods to overcome the poor adhesion between GNs and polymers.

## 1. Instruction

Graphene, a single layer of sp^2^ hybridized carbon atoms packed densely in a two-dimensional honeycomb crystal lattice, has attracted tremendous attention because of its novel properties and potential application in many technological fields such as polymer composites, supercapacitors, lithium batteries, and sensors [1,2,3]. It was suggested that graphene can be used as a nanofiller for carbon filler-based polymer composites as a result of its good chemical stability, huge specific surface area, electrical properties, and remarkable mechanical [4,5,6]. Because of these features, graphene sheets (GNs) can be used as a nanofiller to improve the mechanical, electrochemical, and thermal properties of graphene-based polymer nanocomposites [7,8,9]. For instance, Ni and coworkers successfully synthesized three-dimensional graphene skeleton (3DGS)/epoxy composites by directly mixing 3DGS and epoxy. The compressive strengths increased by 148.3% and the glass transition temperature increased by 19 °C [10].

However, pristine GNs are unsuitable for intercalation by large species, because graphene as a bulk nano-material aggregated an obvious trend in the polymer matrix. The covalent and non-covalent functionalization of graphene may be an effective method to promote the dispersion and stabilization of graphene to prevent agglomeration [11]. The small molecules or polymer chains can be attached to graphene by a covalent modification method. The chemical functionalization of graphene is a particularly attractive target because it can improve both solubility and processability, as well as enhance the interactions with organic polymers [12,13]. Much work has been carried out on amination, isocyanate modification, and polymer wrapping as routes for the modification of graphene. For example, Yang et al. [14] used a facile covalent functionalization method to obtain polydisperse, functionalized, chemically converted graphene nanosheets (f-CCG). They demonstrated that the compressive failure strength and the toughness of f-CCG-reinforced monoliths at 0.1 wt %, compared with the neat monolith, were greatly improved by 19.9% and 92%, respectively. Wan et al. [15] reported the fracture toughness and tensile strength of the bisphenol-A functionalized graphene oxide(GO)/epoxy composites increased by 41% and 75% at 0.5 wt % filler contents, respectively, compared to the neat epoxy. Due to the dispersion and compatibility of GO nanosheets in epoxy, can be effectively improved by the surface functionalization of GO. Yan et al. [16] used the silanization reaction to introduce the imidazole ionic liquid (IMD-Si) onto the surface of GO nanosheets. The addition of 0.4 wt % IMD-Si@GO, leads to increases in flexural strength, flexural modulus, and impact strength of IMD-Si@GO/epoxy composites by 12%, 26% and 52%, respectively, compared to the neat epoxy. However, the chemical reaction modification methods are complex and not easy to control. In recent years, noncovalent modification has become a hot spot of modified graphene. Teng et al. [17] used GNs to absorb the functional segmented poly(glycidyl methacrylate) (Py-PGMA) through π–π stacking and the thermal conductivity of Py-PGMA-GNS/epoxy composite increased by more than 800% with low GNS loading. Liu et al. synthesized a thermosensitive polymer PNIPAm with a tantalum termination and adhered to the graphene surface by π–π stacking. The non-covalently modified PNIPAM-GNs has a lower dissolution temperature than the covalently modified PNIPAM-GS. [18]. Stankovich et al. [19] prepared poly(sodium 4-styrenesulfonate) (PSS) PSS-modified graphene after reduction by van der Waals interactions. Due to the adhesion of PSS, graphene can be stored in an aqueous solution for a long time without agglomeration.

Epoxy resin (EP) has been applied in many fields because of its excellent properties, such as chemical resistance, low shrinkage, and high modulus [20,21,22]. However, EP has a critical defect: it is brittle in nature and therefore has poor impact strength, which greatly limits the applications of EP. As a result strengthened and toughened EP has enjoyed extensive attention. Sue et al. [23] selected α-zirconium phosphate nanoplatelets, which are synthetic crystals with an analogous structure to natural clay platelets, to reinforce epoxy matrix. The epoxy nanocomposite films are transparent and flexible and exhibit an exceptionally high tensile modulus and strength. In our previous work, microcrystallinecellulose fibers (MCFs), were modified by hyperbranched liquid crystals (HLP) to improve the wettability and interactions between the MCFs and epoxy resin [24].

In this paper, liquid crystalline polyurethane modified GNs (GPLP) were obtained by π–π stacking interactions between GNs and perylene bisimide derivatives, and then the GNLP/EP composites were prepared via a casting molding method after the in-situ polymerization of liquid-crystalline polyurethane on the surface of GNs. The introduction of the liquid crystal units endow the GNLP/EP composites with excellent mechanical and thermal properties, due to the enhanced interactions between the GNs and EP.

## 2. Materials and Methods

### 2.1. Materials

4,4′-dihydroxybiphenyl was obtained from Beijing Yangcun Chemical Co., Ltd., (Beijing, China). 6-Chlorohexanol was purchased from Tianjin Damao Chemical Reagent Company, (Tianjin, China). Graphene nanosheets (lateral sizes was 0.5~3 μm and thicknesses was 0.55~3.74 nm) were provided by Ningbo Institute of Industrial Technology of the CAS, (Ningbo, China). Tris(hydroxymethyl)methyl aminomethane, *N*-Methyl pyrrolidone (NMP), Toluene-2,4-diisocyanate (TDI), and 3,4,9,10-Perylene tetracarboxylic anhydride (PTCDA) were purchased from Xiya Chemical Reagent Company, (Chengdu, China). Epoxy resin (E-44, epoxide value was 0.44) was purchased from Yueyang Chemical Plant, (Yueyang, China). Zinc acetate anhydrous used as the latentcatalyst and 4,4′-Diaminodiphenylsulphone (DDS) used as the curing agent were purchased from Sinopharm Chemical Reagent Co., Ltd., (Shanghai, China). The solvent DMF and acetone were purchased from Guanghua Sci.Tech. Co., Ltd., (Guanghua, China). Tris(hydroxymethyl)methyl aminomethane (THAM), KI and KOH were purchased from Aladdin.

### 2.2. Synthesis of Biphenylnate Liquid Crystalline (BP6)

**Scheme 1:**
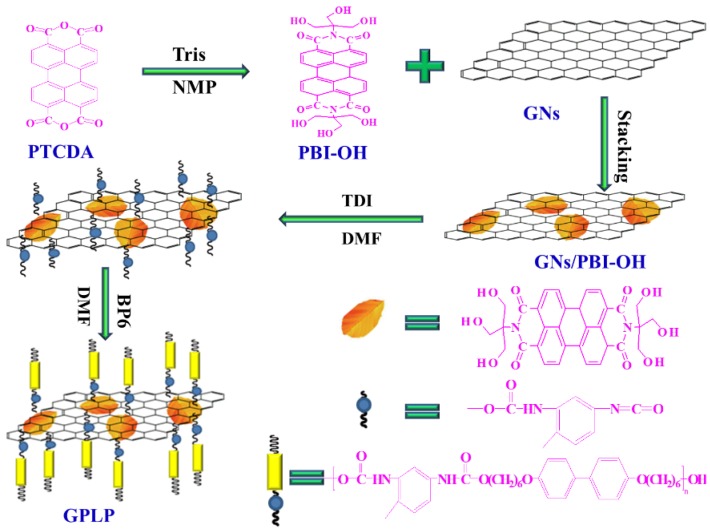
The preparation process of the GNs/PBI-OH/Liquid Crystalline Polyurethane (GPLP).

BP6 was synthesized by a nucleophilic substitution reaction of 4,4′-dihydroxybiphenyl and 6-Chlorohexanol according to Scheme 1. Firstly, 3.72 g (0.02 mol) 4,4′-dihydroxybiphenyl and 2.24 g (0.04 mol) KOH and small traces of KI were dissolved in a 250 mL round-bottom with moderate pure alcohol, and reacted for 2 h at room temperature. After that, 5.46 g (0.04 mol) 6-Chlorohexanol was added to the solution and refluxed for 24 h. The cold mixture was precipitated into water, washed, and filtered with deionized water. The resulting white product was recrystallized from alcohol.

### 2.3. Synthesis of the Perylene Bisimide Derivatives

PTCDA (3.92 g, 0.01 mol), Tris(hydroxymethyl)methyl aminomethane (9.68 g, 0.08 mol), Zinc acetate anhydrous(1.83 g, 0.01 mol), and 100 mL NMP were added to a three-neck flask equipped with a nitrogen protective device. The mixture reacted at 160 °C for 16 h under constant stirring. We found that the color of the mixture solution became red brown from red gradually as the reaction progressed. The mixture solution was added to a beaker with 600 mL alcohol heated to 60 °C under constant stirring, then filtered and washed with hot alcohol. At last, the red brown perylene bisimide derivatives (PBI-OH) were dried under vacuum.

### 2.4. Preparation of GNs/PBI-OH/Liquid Crystalline Polyurethane (GPLP)

As shown in Scheme 1, the GPLP was prepared as follows: First, PBI-OH (0.2 g, 0.4 mmol), equal quality of GNs and 100 mL DMF were added to a three-neck round-bottom flask, and then the mixture underwent ultrasonic treatment for 1h. After that, TDI (2.09 g, 0.012 mol) and catalytic amounts of Dibutyltin dilaurate were added. The resulting mixture was refluxed at 80 °C for 8 h under nitrogen protection. Second, BP6 (5 g, 0.02 mol) was dropped into the mixture slowly (5–10 drops per minute) after it dissolved in 60 mL DMF. The system was kept at 80 °C and reacted under constant stirring for another 8 h. The resulting mixture was precipitated in water, filtered, and washed with deionized water. The precipitated purple-black was put into a large beaker with moderate alcohol, heated to boiling, then filtered and washed with hot alcohol to remove the excess BP6 and polyurethane oligomer. Finally, the solid product (GPLP) was dried under vacuum. 

### 2.5. Preparation of Epoxy/GPLP Composites

**Scheme 2:**
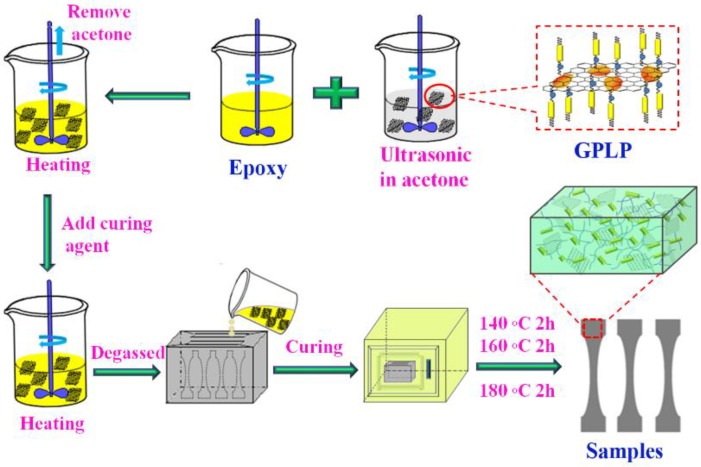
The preparation process of the epoxy/GPLP composites.

The epoxy/GPLP composite was prepared as follows: First, a certain amount of GPLP was dispersed in acetone for 30 min by ultrasonic. At the same time, 26 g epoxy was added to a beaker, stirred and degassed to remove little water at 80–90 °C. Second, the resulting GPLP suspension was slowly added to the epoxy. The mixture was stirred until the GPLP was uniformly scattered, and then heated to 60 °C to remove the acetone. Third, 7.8 g DDS was added to the resulting mixture under vigorous stirring. Finally, the resulting mixture was poured into a preheated mold and degassed under vacuum for 30 min, then cured at 140 °C for 2 h, 160 °C for 2 h, and 180 °C for 2 h to get the samples as shown in Scheme 2. The different weight fractions of GPLP were 0.0, 0.5, 1.0, 2.0, 3.0, 4.0 wt % were prepared. According to the hermo-gravimetric analyzer analysis, the graphene content in the GPLP was 49%. Therefore, the different contents of GNs were 0.0, 0.245, 0.49, 0.98, 1.47, 1.96 wt % were prepared.

### 2.6. Characterization

Nicolet Nexus 470 (Nicolet Corporation, Madison, VA, USA) was used to record the Fourier-transform infrared spectra (FTIR) ranging from 400 to 4000 cm^−1^. The liquid crystal properties of all the samples were examined by using a polarizing microscope equipped with a Nikon Fm10 camera (Nikon Fm10, Nikon Cooperation, Tokyo, Japan) and an INSTEC heating stage (INSTEC, Rui Tong Electrical and Mechanical Equipment Co., Ltd., Shanghai, China), and the heating rate was 5 °C/min. A Holland PAN-analytical X-Pert PRO X-ray diffractometer (PANalytical B.V., Almelo, Holland) was used to record the X-ray diffraction (XRD) patterns. The ESCALAB 250Xi instrument (Thermo Electron Corporation, Waltham, MA, USA) was used to recorded the X-ray Photoelectron Spectroscopy (XPS). UV-Vis spectra were recorded on a spectrophotometer (UV 3600 SHIMADZU Company, Kyoto, Japan) with the wavelength ranging from 200 to 800 nm, and all the samples were dispersed in alcohol. Differential scanning calorimetry spectrometer (DSC-204, Netzsch, Wittelsbacherstr, Germany) was used to study the melting and clearing point temperature of liquid crystal and the *T*_g_ of epoxy/GPLP composites with a heating rate of 10 °C/min. TGA Q-500 thermo-gravimetric analyzer (TGA Q-500, TA Instrument, Brussels, Belgium) was used to test the thermal decomposition temperatures of samples. A field emission scanning electron microscope (FE-SEM, JSM-6701F, Tokyo, Japan) was used to observe the morphology of samples and a thin layer of gold was sputtered on the fracture surfaces. A dynamic mechanical analyzer (Q-800, TA Instrument, New Castle, DE, USA) was used to characterize the storage modulus of the epoxy/GPLP composites in the single cantilever mode with a frequency of 1 Hz from 30 to 250 °C. A tester of type XJJ-5 (Chengde Precision Testing Machine Co., Ltd., Chengde, China) was used to evaluate the impact strength of the epoxy/GPLP composites according to the National Standard of China (GB1043-79). The tensile strength and flexural strength were evaluated on a universal tensile tester (Jinan Tianchen Testing Machine Manufacturing Co., Ltd, Jinan, China) according to National Standard of China (GB1040-92) at the rate of 2 mm/min. The shape of the specimen is dumbbell-shaped, 5 mm wide and 2 mm thick for tensile testing, while the specimens for the three-point static bending test were rectangular with the size of 80 mm × 10 mm × 4 mm.

## 3. Results and Discussion

### 3.1. Characterization of the BP6, PBI-OH, and GPLP

**Figure 1:**
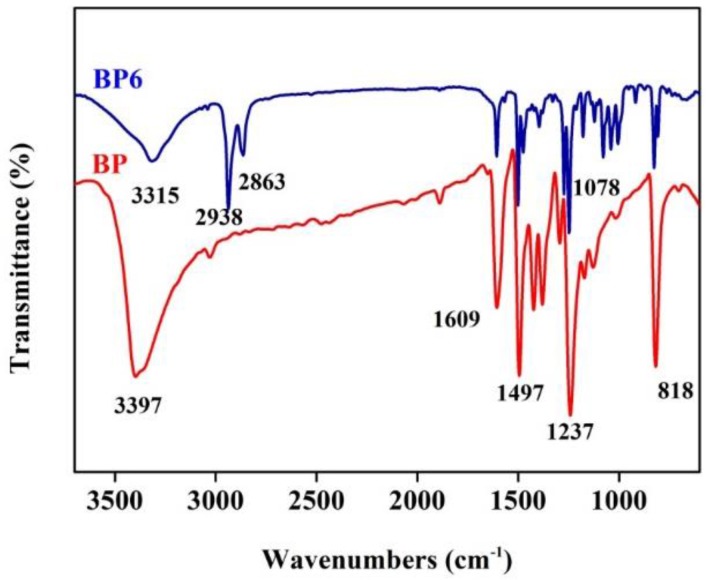
the Fourier-transform infrared spectroscopy (FTIR) spectra of BP and BP6.

As shown in Figure 1, a strong absorption peak of phenol hydroxyl groups (–OH) at 3397 cm^−1^, the absorbance band of aromatic C=C bonds at 1609 cm^−1^, ether bond C–O–C absorption at 1078 cm^−1^, 1497, and a C–O stretching vibration band at 1237 cm^−1^ appeared in the BP spectrum. In the spectra of BP6, the peak shift of –OH from 3397 to 3315 cm^−1^ was attributed to the nucleophilic substitution reaction of 6-Chlorohexanol, the phenol hydroxyl group has changed to an alcoholic hydroxyl group. Additionally, several new peaks, such as aliphatic C–H group symmetric stretching vibration and anti-symmetric stretching vibration at 2938 and 2863 cm^−1^ appeared in the spectra of BP6. These results indicated that liquid crystal monomer BP6 had been successfully synthesized.

**Figure 2:**
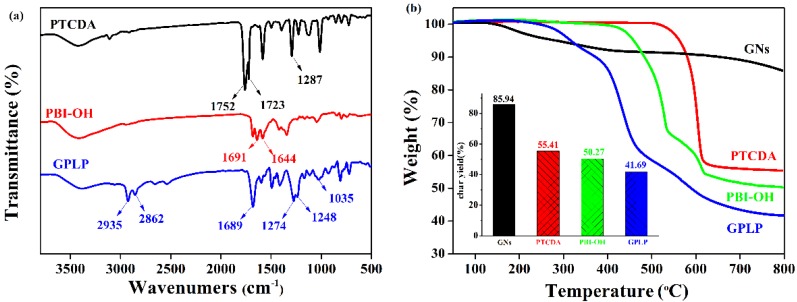
(**a**) FTIR spectra; and (**b**) Thermo-gravimetric analyzer (TGA) curves of graphene nanosheets (GNs), Perylene tetracarboxylic anhydride (PTCDA), PBI-OH and GPLP.

In Figure 2a, the FTIR spectrum of PBI-OH exhibited two characteristic absorption peaks at 1752 and 1723 cm^−1^, which was attributed to a coupling band of perylene anhydride C=O stretching vibration. In the PBI-OH spectrum, however, the characteristic absorption peaks of perylene anhydride C=O stretching vibration disappeared and the band of imide C=O appeared at 1691 and 1644 cm^−1^, indicating that the amidation reaction between PTCDA with THAM was formed PBI-OH. Compared to the spectra of GNs and PBI-OH, the FTIR spectrum of GPLP exhibited two strong specific absorbing peaks at 1274 and 1248 cm^−1^, which were assigned to –COOC stretching. According to the condensation polymerization of PBI-OH, TDI, and BP6, the absorbance bands of –CH_2_– at 2935 and 2862 cm^−1^ were observed. The broad absorption band at 3391 cm^−1^ can account for the overlay of N–H and –OH stretching vibration. 

As shown in Figure 2b, the thermal stability of PTCDA, GNs, PBI-OH, and GPLP were studied by TGA. It can be seen that GNs remain stable over the range of test temperature, and the char yield was 86%. A certain level of decomposition was detected over 300 °C, due to the loss of segments of the alkyl chain. In contrast to GNs and PBI-OH, the TGA curve of GPLP showed a two step decomposition. One decomposition at 300 °C is assigned to the flexible chain at the end of the liquid crystalline polyurethane. The other decomposition at 400 °C is assigned to the rigid segments of liquid crystalline polyurethane and the char yield of GPLP was 42%. Therefore, according to the TGA analysis the graphene content in the GPLP was about 49%. This suggests that the GNs were successfully modified by liquid crystalline polyurethane (LCP) and further validates the FTIR findings. 

**Figure 3:**
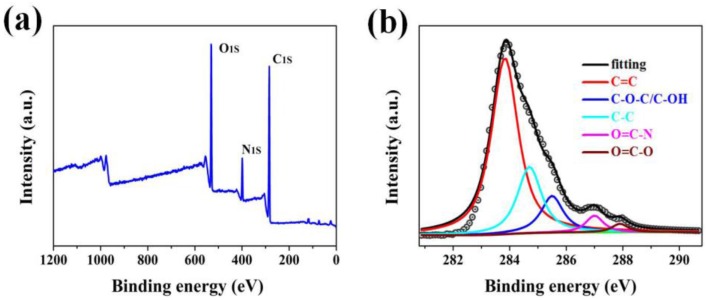
X-Ray spectroscopy (XPS) survey spectra of (**a**) GPLP; and (**b**) C1s XPS spectrum of GPLP.

As shown in Figure 3, the chemical nature of GPLP was quantitatively elucidated by XPS. In Figure 3a, a peak at 399 eV corresponds to N 1s, which was ascribed to the modification of PBI/LCP and the O/C atomic ratio of GPLP was calculated as 1.05. Furthermore, the high resolution C 1s spectrum of GPLP shows the components that correspond to the different functional groups: graphitic carbon, hybridized carbon, ether, hydroxyl, ester group, and amido (as shown in Figure 3b). The five carbon species were determined at specific Binding Energy, i.e., hybridized carbon (C–C sp^3^, 284.9 eV), graphitic carbon (C=C sp^2^, 283.8 eV), ether and hydroxyl (C–O–C/C–OH, 285.6 eV), ester group (O=C–O, 287.9 eV), and amido (O=C–N, 287.2 eV) [25]. These results are in agreement with FTIR characterization and confirm that the surface of GNs was modified by LCP.

**Figure 4:**
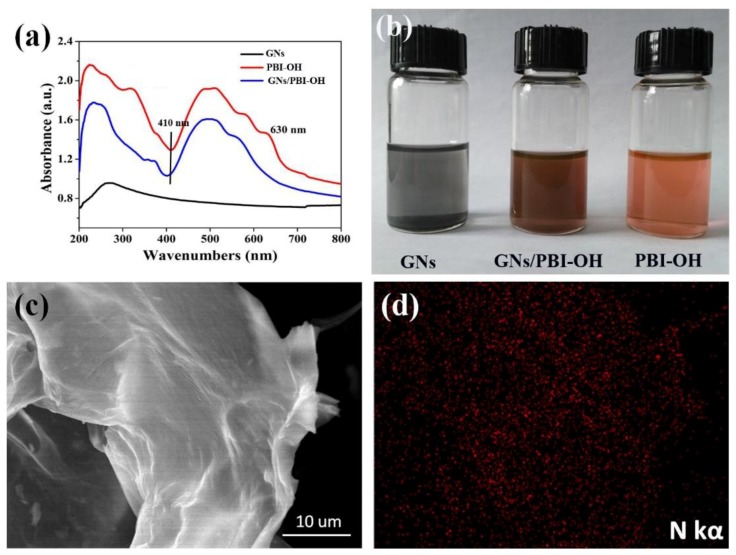
(**a**) UV-Vis spectra of GNs, PBI-OH and GNs/PBI-OH; (**b**) Solubility of GNs, PBI-OH and GNs/PBI-OH in DMF; (**c**) The scanning electron microscope (SEM) image of GNs/PBI-OH; and (**d**) Elemental mapping of N is shown in red.

The UV-Vis absorption spectra of GNs, PBI-OH and GNs/PBI-OH were presented in Figure 4a. The UV-Vis spectrum of PBI-OH showed four characteristic absorption peaks assigning to the different n–π* electronic transitions above 250 nm. However, the absorption peak at 630 nm in the spectra of GNs/PBI-OH disappeared and the peak at 410 nm generated a blue shift. This is mainly because the PBI-OH was adsorbed on the surface of the GNs sheets after π–π stacking and influenced by the basis of the quasi-molecular electronic structure of the sp^2^ clusters of GNs [26,27]. Therefore, the groups on the PBI-OH may dissipate their excited state energy to the surrounding sp^3^ structure, and therefore lead to the blue shifting and low emission intensity [28]. As shown in Figure 4b, the PBI-OH dissolved well in polar solvent DMF, but the GNs precipitated from DMF. However, the GNs can also disperse steadily into DMF by ultrasonic treatment with equal quality PBI-OH for 1h, which proved that PBI-OH was adsorbed on the surface of the GNs sheets via π–π stacking force. The SEM image of GNs/PBI-OH displayed a wrinkled GNs sheet and N elemental was evenly distributed across all the partitions of GNs sheet as shown in Figure 4c,d. Combined with the XPS analysis of GNs, the results indicate that the PBI-OH adheres better to GNs sheets.

**Figure 5:**
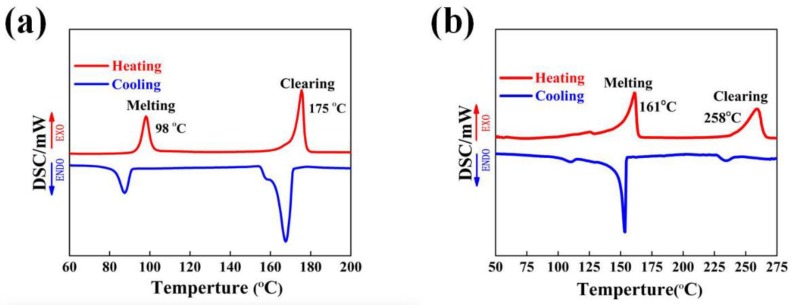
Differential scanning calorimetry (DSC) curves of BP6 (**a**) and GPLP (**b**).

Differential scanning calorimetry (DSC) was used to study the thermal properties of BP6 and GPLP and their phase transition temperatures obtained on the second heating and the first cooling scan were displayed in Figure 5. As shown in Figure 5a, the DSC heating thermogram of BP6 contained two endotherms of the phase transition, at 98 °C was a melting transition and at 175 °C was a nematic-isotropic phase transition of BP6. The temperature interval between the two phase transitions indicate the extent of the liquid crystalline phase region. As shown in Figure 5b, two peaks corresponding to the endotherms of the phase transition at 161 and 258 °C appeared in the DSC curves of GPLP, which shifted to a higher temperature duo to the polymerization and the introduction of hard segment perylene. It was indicated the phase transition from the mesophase to the isotropic phase of GPLP requires more energy. The optical textures of the two samples were further investigated by polarizing microscope (POM) with hot stage. 

**Figure 6:**
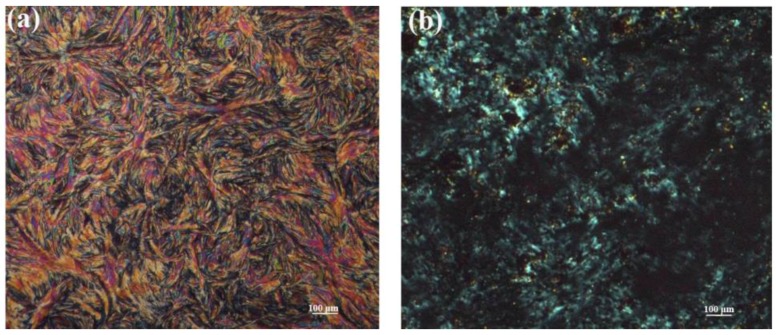
Polarizing microscope (POM) of the BP6 (**a**) and GPLP (**b**).

As shown in Figure 6a, a well-developed texture of BP6 was presented as a representative sample. The BP6 was characterized by white crystals at ambient temperature. When the temperature was raised to 100 °C, the white crystals began to melt and the fingerprint texture was well-distributed evenly with increasing temperature. Once the temperature was over 180 °C, the bright stripes disappeared and the field of vision became dark. Figure 6b shows the polarized optical micrograph of the texture of GPLP, which is in the shape of a fan between the temperature of the melting transition and a nematic-isotropic phase transition. In contrast to BP6, the GPLP was less liquid and poor liquid crystal. That is because of the influence of GNs with a larger specific surface area, which limits the liquid of GPLP. 

### 3.2. Thermal Dynamic Properties of Epoxy/GPLP Composites

**Figure 7:**
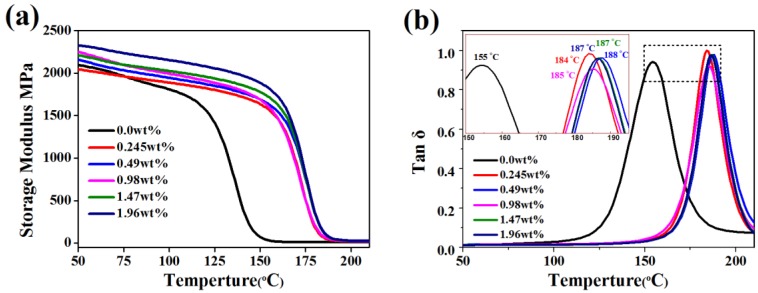
(**a**) The storage modulus; and (**b**) tan d of epoxy/GPLP composites at 1 Hz frequency.

As shown in Figure 7, storage modulus and the glass transition temperature (*T*_g_) of epoxy/GPLP composites were investigated by Dynamic mechanical analysis. The crosslink density of the epoxy/GPLP composites was calculated using an expression based on Flory’s rubber elasticity theory [29,30]:

(1) $$V_{e}=E^{\prime }/RT$$

where *V*_e_ is the crosslink density expressed in moles per cubic centimeter of a sample, *E*’ is the storage modulus in the rubbery region at a temperature well above the glass transition temperature, *R* is the universal gas constant and the value is 8.31 J·K^−1^·mol^−1^, and *T* is the absolute temperature corresponding to the storage modulus *E*′ selected. The results were calculated on base of DMA analysis and listed in Table 1. Storage modulus of epoxy/GPLP composites were higher than that of neat epoxy by incorporating with the GPLP during the whole test. In addition, the room storage modulus was enhanced gradually, with the consumption of GPLP in the thermosets. When the content of GNs reached 1.96 wt %, the materials achieved a higher storage modulus about 2325 MPa at 50 °C, enhanced by 228 MPa compared to the 2097 MPa of neat epoxy. The *T*_g_ values were measured from the peaks of the tanδ curves as presented in Figure 7b and Table 1. The *T*_g_ of neat epoxy and its composites with 1.96 wt % GNs were 155 °C and 185 °C, respectively, which increased by 30 °C. The tan δ curves of composites shifted to a higher temperature after filling with a larger amount of GPLP. These results can be attributed to three reasons. First, the GNs sheets have a higher modulus and act as physical interlock points, which reduces the chains mobility. Second, the entanglements formed by liquid crystal polyurethane chains also reduce the chains mobility. Last, but not least, the crosslink density increases with an increase in the GPLP amount, molecular motions see an amount of energy and could be more restricted during the continuous increase of the temperature [31].

**Figure 8:**
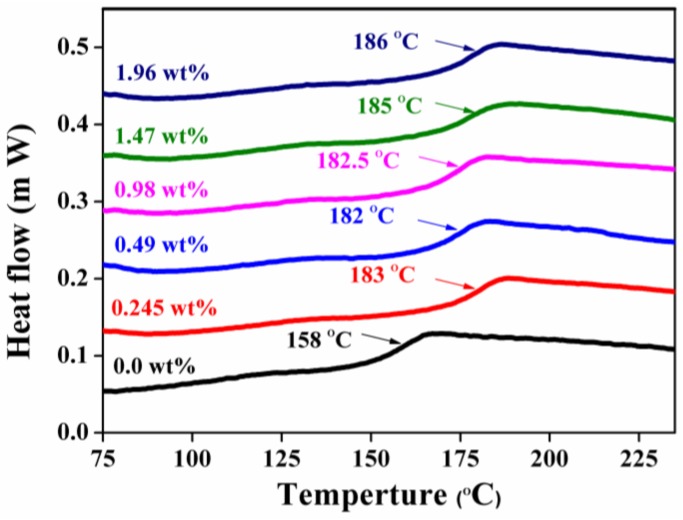
DSC curves of epoxy/GPLP composites.

The *T*_g_ of epoxy/GPLP composites were recorded by DSC and the DSC curves of samples with different loadings of GPLP were presented in Figure 8. It can be seen that the *T*_g_ of neat epoxy was about 158 °C, which is lower than that of epoxy/GPLP composites filled with GPLP. Meanwhile, the *T*_g_ shift to high values with the adding of GPLP into epoxy matrix, and the highest value 186 °C was assigned to the composite filled with 1.96 wt % GNs, increasing by 28 °C compared to that of neat epoxy. This phenomenon can be attributed to the movement of molecular a fragment and was impeded by the GNs, which acted as physical interlock points in the epoxy matrix. What is more, the improvement of crosslink density also played an important role in restraining the chain mobility.

### 3.3. The Morphologies of the Fracture Surfaces of Epoxy/GPLP Composites

**Figure 9:**
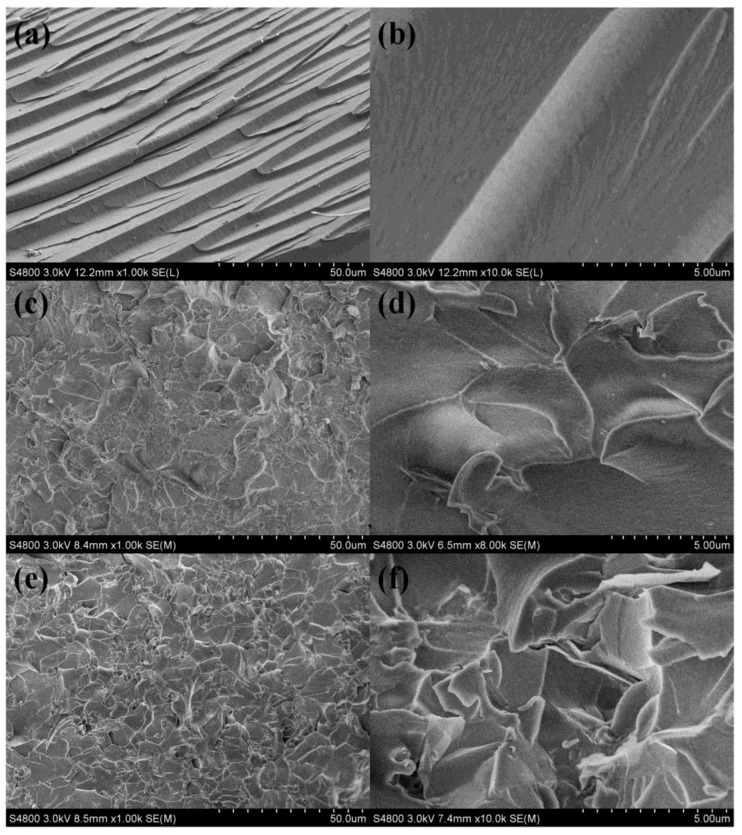
SEM images of fractured surface of composites with different enlargement fractions: (**a**,**b**) neat epoxy, (**c**,**d**) 0.49 wt % GNs, (**e**,**f**) 1.96 wt % GNs.

As shown in Figure 9, the morphologies of the fracture surfaces of the neat epoxy and its composites with magnification were recorded by SEM. In Figure 9a,b, it can be seen that the fracture surface of EP was smooth and similar to river patterns. In addition, the crack propagation directions are consistent with the impact stress, which indicates that the material was broken by brittle fracture [32]. As shown in Figure 9e,f, however, the fracture surfaces of the epoxy/GPLP composites exhibited a rougher fracture surface with numerous gullies and wrinkles, especially with 1.96 wt % GNs. Moreover, the GNs cannot be separated from the epoxy matrix, but were embedded with the epoxy matrix. The good interface interaction between GNs and EP was mainly attributed to liquid crystal polyurethane chains and the higher curing temperature, which exceeded the melting point of the liquid crystal polyurethane chains. It is worth a reminder that the interface cracks, which are mixed ones that eventually concentrate on different wrinkle areas, do not always propagate in a single direction. The propagation of cracks can be inhibited by GNs sheets [33]. When microcracks encounter GPLP components, the fracture must bypass them and seek alternative routes, and thus form ductile fracture zones, which consume more energy in the breaking process. Therefore, the breaking strength of the epoxy/GPLP composites was improved by combining with GPLP [34].

### 3.4. Thermal Properties of Epoxy/GPLP Composites

**Figure 10:**
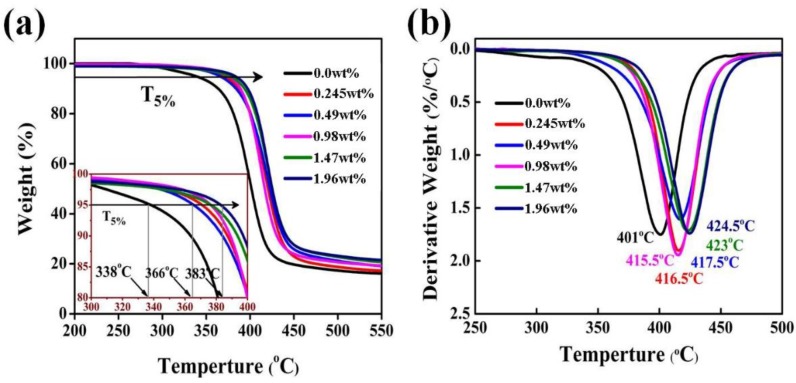
(**a**) TGA curves; and (**b**) DTG curves of epoxy/GPLP composites.

The thermal properties of the neat epoxy and its composites were conducted on TGA at a heating rate of 10 °C/min under nitrogen atmospheres. As shows in Figure 10, the neat epoxy and its composites exhibited similar thermal decomposition behavior with one-step decomposition, which indicated the addition of GPLP did not change the degradation mechanism of epoxy resins. In Figure 10b, however, it can be seen that the epoxy/GPLP composites exhibited higher thermal stability. The initial degradation temperature (*T*_5%_) at 5% weight loss (*T*_5%_) of epoxy/GPLP composites was over 366 °C and the maximum *T*_5%_ of epoxy composite was up to 383 °C by the introduction of 4% GPLP. compared with the 338 °C of neat epoxy resins, the *T*_5%_ of epoxy composite increased by about 45 °C. Moreover, the maximal degradation temperature (*T*_max_) of epoxy/GPLP composites increased from 401 to 424.5 °C, which was 23.5 °C higher than that of the neat epoxy resins. The *T*_5%_ and *T*_max_ are listed in Table 1. On the one hand, the residual hydroxyl and amino of the surface of GPLP and the epoxy matrix reacted to produce a chemical crosslink [35]. On the other hand, the liquid crystal polyurethane chains formed physical entanglements with the epoxy resins during the curing process. Therefore, the improvement of crosslink densities enhanced the themostability of the epoxy/GPLP composites. In addition, it was found that the char yield of the epoxy/GPLP composites are higher than that of neat epoxy at 550 °C, and char yield increased with the increasing of GPLP contents, which can be attributed to the GNs very stable condition under 600 °C.

### 3.5. Mechanical Properties of Epoxy/GPLPcomposites

**Figure 11:**
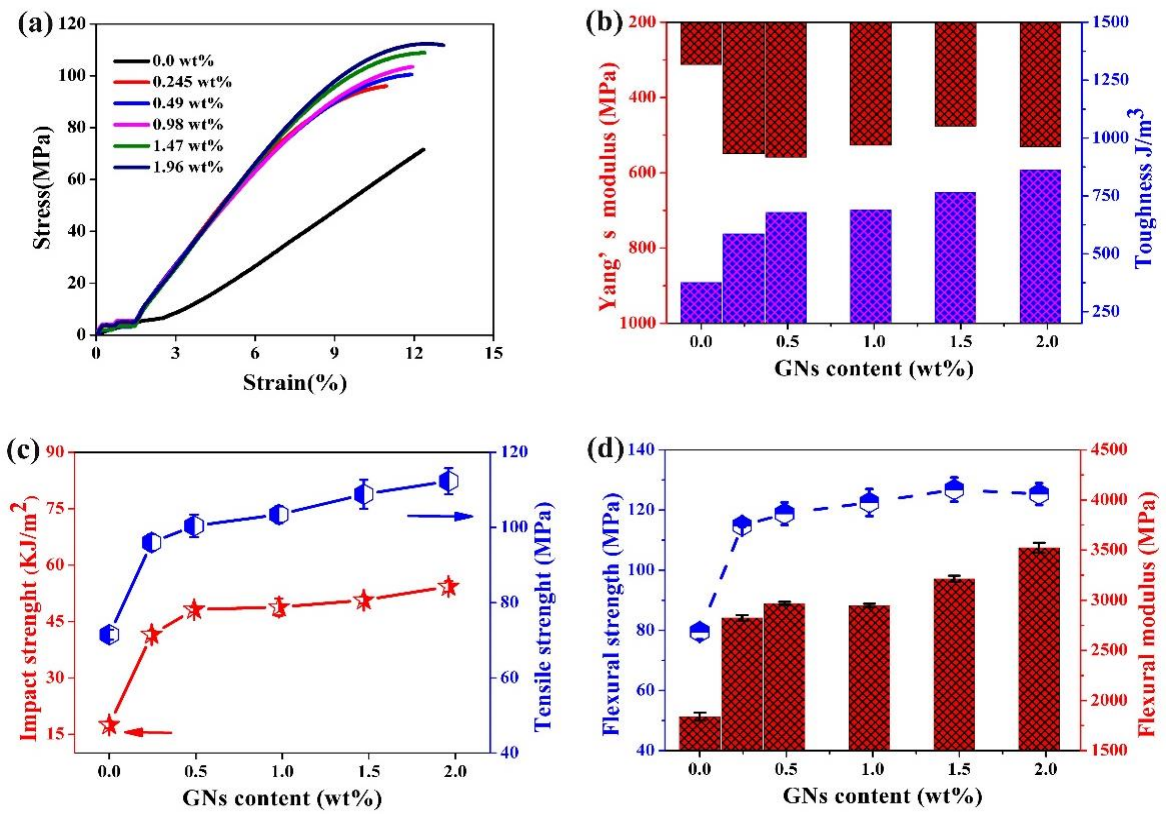
(**a**) typical curves of stress-strain; (**b**) Young’s modulus and toughness; (**c**) Impact strength and tensile strength; (**d**) flexural strength and modulus of neat epoxy and its composites.

As shown in Figure 11, the mechanical properties of neat epoxy and its composites with different contents were investigated by a tester of type XJJ-5 and a universal tensile tester. It can be seen from Figure 11a the stress-strain curve of neat epoxy exhibited linear stress-strain character up to failure without plastic deformation. However, the curves of the epoxy/GPLP composites produced a little plastic deformation over the elongation of 9%, and the amount of deformation increase with the improvement of the percentage of GPLP. The toughness and Yang’s modulus of epoxy/GPLP composites were evaluated directly by means of the stress-strain curves shown in Figure 11a and the results were presented in Figure 11b. It is obvious that the toughness of the epoxy/GPLP composites were enhanced gradually in conformity with the content of GPLP. When the filling content of GNs was 1.96 wt %, the value for the toughness was 863 J/m^3^, increasing by 122% compared the 376.5 J/m^3^ of neat epoxy. Similarly, the Yang’s modulus was also improved by the participation of modified GNs. For instance, the epoxy/GPLP composites with 1.96 wt % GNs exhibited a Young’s modulus of 530.8 MPa, which increased by 218.6 MPa in contrast to the value of pure epoxy. 

Figure 11c shows the changing trends of impact strength and tensile strength of epoxy/GPLP composites according to GNs content variations. The results are summarized in Table 2. Apparently, the changing trends of impact strength and tensile strength are very similar according to GNs content variations from Figure 11c: the impact strength and tensile strength improve rapidly when the content of GNs is below 0.49 wt %, and yet the strength showed a more moderate pace between 0.49 and 1.96 wt %. This phenomenon is attributed to the dramatic promotion of crosslink density as listed in Table 1. The maximum values of impact strength and tensile strength corresponding to epoxy/GPLP composites with 1.96 wt % GNs were 54.31 kJ/m^2^ and 112.33 MPa, which increased by 210% and 57% compared to that of neat epoxy, respectively. As shown in Figure 11d and Table 2, the flexural strength and flexural modulus of the epoxy/GPLP composites also significantly improved. The flexural strength values of epoxy/GPLP composite with different GNs loadings range from 114.8 to 125.3 MPa, which were much higher than the 79.5 MPa of neat epoxy. The change trend of the flexural modulus is similar to the curves of impact strength and tensile strength, the maximum of the epoxy composite with 1.96 wt % GNs was about 3.5 GPa, which increased by 91.7% compared to that of neat epoxy (1.8 GPa). At 1.47 wt % GNs contents, the tensile strength and impact strength of epoxy/GPLP composites were 50.68 kJ/m^2^ and 108.84 MPa, which increased by 170% and 79% compared to that of epoxy/GNs composite, respectively (Figure S1). 

## 4. Conclusions

In this study, liquid crystal polyurethane modified graphene nanosheets were prepared by π–π stacking interactions method. The results from FTIR, TGA, SEM and XPS indicate the successful preparation of GPLP, and the GNs content was calculated to be 49% from the TGA analysis. The impact strength, tensile strength, Young’s modulus, and toughness of the GPLP/EP composites were 54.31 kJ/m^2^, 112.33 MPa, 530.8 MPa, and 863 J/m^3^, respectively. These values showed significant increases of 210%, 57%, 143%, and 122% when compared to neat epoxy. Additionally, DMA reveals that the glass transition temperature of the GPLP/epoxy composites can be improved by adding very low GPLP loadings. Moreover, a little inclusion of GPLP into EP can significantly improve thermal stability. These enhancements can be attributed to the uniform dispersion of GPLP and strong interfacial interactions with the EP matrix. It is expected that this work could provide an effective method for improving the interactions between GNs and a polymeric matrix.

## Figures and Tables

**Table 1:** the thermo-mechanical properties of epoxy/GPLP composites and crosslink density (*V*_e_).

Specimen Category	Storage Modulus, *E* (MPa) at 50 °C	Glass Transition Temperature, *T*_g_ (°C)	Storage Modulus, *E*′ (MPa) at *T*_g_ + 50 °C	Crosslink Density, *V*_e_ (mol/m^3^)
0.0 wt %	2097	155	10.16	852.2
0.245 wt %	2046	185	21.74	1715.8
0.49 wt %	2158	184	22.98	1810.9
0.98 wt %	2254	187	25.39	1996.0
1.47 wt %	2210	187	26.97	2120.2
1.96 wt %	2325	188	28.79	2258.9

**Table 2:** The mechanical properties of neat epoxy and epoxy/GPLP composites.

GNs Content	Impact Strength (kJ/m^2^)	Tensile Strength (MPa)	Flexural Strength (MPa)	Flexural Modulus (MPa)
0.0 wt %	17.50 ± 0.92	71.51 ± 1.33	79.5 ± 2.5	1840 ± 40
0.245 wt %	41.53 ± 1.16	96.00 ± 2.21	114.8 ± 0.3	2825 ± 30
0.49 wt %	48.19 ± 0.75	100.45 ± 2.99	118.8 ± 3.8	2970 ± 15
0.98 wt %	48.89 ± 2.26	103.45 ± 2.23	122.5 ± 4.5	2947 ± 18
1.47 wt %	50.68 ± 0.92	108.84 ± 3.83	126.8 ± 4.0	3214 ± 32
1.96 wt %	54.31 ± 1.41	112.33 ± 3.47	125.3 ± 3.6	3525 ± 50

## Supplementary Materials

The following are available online at http://www.mdpi.com/2073-4360/10/5/485/s1, Figure S1: (a) Impact strength and tensile strength of EP, EP/GNs and (b) EP/GPLP composites (GNs 1.47 wt %).
