# Supplementary material for: High Mechanical and Thermal Properties of Epoxy Composites with Liquid Crystalline Polyurethane Modified Graphene
Source: Polymers (Basel). 2018 May 1;10(5):485. doi: 10.3390/polym10050485 (PMC6415506; doi:10.3390/polym10050485)
Supplement: Supplementary file 1 [file polymers-10-00485-s001.pdf]

# High mechanical and thermal properties of epoxy composites with liquid crystalline polyurethane modified graphene

**Yuqi Li*, Jian Gao#, Xiuyun Li, Xu Xu and Shaorong Lu***

Key Laboratory of New Processing Technology for Nonferrous Metals and Materials, Ministry of Education, College of Materials Science and Engineering, Guilin University of Technology, Guilin 541004, China.

*Correspondence: liyuqi@glut.edu.cn; lushaor@163.com

#These authors contributed equally to this work.

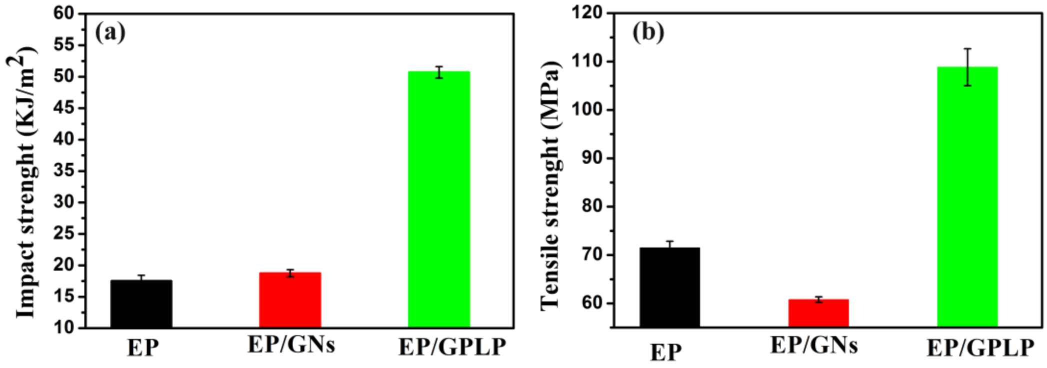


Figure s1 (a) Impact strength and tensile strength of EP, EP/GNs and (b) EP/GPLP composites (GNs 1.47 wt %).
